# Comparative Study on the Essential Oils from Five Wild Egyptian *Centaurea* Species: Effective Extraction Techniques, Antimicrobial Activity and In-Silico Analyses

**DOI:** 10.3390/antibiotics10030252

**Published:** 2021-03-03

**Authors:** Eman H. Reda, Zienab T. Abdel Shakour, Ali M. El-Halawany, El-Sayeda A. El-Kashoury, Khaled A. Shams, Tarik A. Mohamed, Ibrahim Saleh, Abdelsamed I. Elshamy, Mohamed A. M. Atia, Ahmed A. El-Beih, Nahla S. Abdel-Azim, Hesham R. El-Seedi, Mohamed-Elamir F. Hegazy

**Affiliations:** 1 Phytochemistry Laboratory, National Organization for Drug Control and Research, Giza 12622, Egypt; dremanhusseinreda@gmail.com (E.H.R.); zizishakour@yahoo.com (Z.T.A.S.); 2 Department of Pharmacognosy, Faculty of Pharmacy, Cairo University, Cairo 11562, Egypt; ali.elhalawany@pharma.cu.edu.eg; 3 Chemistry of Medicinal Plants Department, National Research Centre, 33 El-Bohouth St., Dokki, Giza 12622, Egypt; ka.saad@nrc.sci.eg (K.A.S.); ta.mourad@nrc.sci.eg (T.A.M.); ia.saleh@nrc.sci.eg (I.S.); ns.abdelazim@nrc.sci.eg (N.S.A.-A.); 4 Department of Natural Compounds Chemistry, National Research Centre, 33 El-Bohouth St., Dokki, Giza 12622, Egypt; ai.el-shamy@nrc.sci.eg; 5 Molecular Genetics and Genome Mapping Laboratory, Genome Mapping Department, Agricultural Genetic Engineering Research Institute (AGERI), Agricultural Research Center (ARC), Giza 12619, Egypt; matia@ageri.sci.eg; 6 Chemistry of Natural & Microbial Products Department, National Research Centre, Dokki, Giza 12622, Egypt; aa.el-beih@nrc.sci.eg; 7 Department of Molecular Biosciences, The Wenner-Gren Institute, Stockholm University, S-10691 Stockholm, Sweden; 8 International Research Center for Food Nutrition and Safety, Jiangsu University, Zhenjiang 212013, China; 9 Department of Pharmaceutical Biology, Institute of Pharmaceutical and Biomedical Sciences, Johannes Gutenberg University, Staudinger Weg 5, 55128 Mainz, Germany

**Keywords:** *Centaurea* species, Asteraceae, essential oils, antimicrobial, microwave-assisted extraction, hydro-distillation

## Abstract

The genus *Centaurea* is recognized in folk medicine for anti-inflammatory, anti-itch, antitussive, purgative, astringent, and tonic activities. To study the chemical determinant for antimicrobial activity essential oils (EOs), five *Centaurea* species were analyzed including: *C. scoparia*, *C. calcitrapa*, *C. glomerata*, *C. lipii* and *C. alexandrina*. Conventional hydro-distillation (HD) and microwave-assisted extraction (MAE), as new green technologies, were compared for the extraction of essential oils. GC/MS analysis identified 120 EOs including mostly terpenoid except from *C. lipii* and *C. alexandrina* in which nonterpenoids were the major constituents. Major terpenoids included spathulenol, caryophyllene oxide and alloaromadendrene oxide-2. To probe antibacterial activity, potential EO inhibitors of a bacterial type II DNA topoisomerase, DNA gyrase B were screened via an in silico molecular docking approach. Spathulenol and alloaromadendrene oxide-2 possessed the best binding affinity in the ATP- binding pocket of Gyrase B enzyme. Principal component analysis and agglomerative hierarchical clustering were used for sample classification and revealed that sesquiterpenes contributed the most for accessions classification. In vitro antimicrobial activity against *Staphylococcus aureus*, *Escherichia coli* and *Aspergillus niger* for all EOs were also evaluated. EOs from *C. lipii*, *C. glomerata* and *C. calcitrapa* exhibited significant MIC against *S. aureus* with an MIC value of 31.25 µg/mL.

## 1. Introduction

*Centaurea* is the fourth largest genus within the Asteraceae family, with more than 600 species distributed worldwide, particularly in Western Asia and Mediterranean regions [1,2] About 17 *Centaurea* species are distributed in the Mediterranean coastal areas, the Red Sea, and Nile regions of Egypt. In folk medicine, *Centaurea* species are used as anti-diarrheal (e.g., to increase appetite, gain energy, to relieve chest tightness); febrifuge (e.g., to reduce fever, start menstruation, relieve constipation); an astringent; diuretic; digestive; tonic; expectorant (e.g., for treatment of liver diseases); and as an antipyretic agent [3]. In Egypt, *C. alexandrina* is reported as a remedy for hyperglycemia [4], and the flowering branch extracts are used as anti-bacterial and antidiabetic agents [5]. *C. calcitrapa* is widely used in folk medicine in North Africa; the whole plant is bitter-astringent, appetizer, anti-febrile, stomachic and is used for intermittent fever and eye diseases. In addition, the roots and fruits are diuretic and seeds are used for renal stones [6]. In Syria, the seeds and roots are used as an appetite enhancer and for diarrhea [7]. In Turkey, the plant infusion is used as a febrifuge [8]. Infusion of *C. calcitrapa* L. is used to treat fever, besides being emmenagogue, laxative and appetizer [9].

*Centaurea* shows high structural diversity for its bioactive compounds, including sesquiterpene lactones, triterpenes, flavonoids and lignans [10,11]. *Centaurea* EOs exhibit qualitative and quantitative differences due to the genetic, geographic distribution, local, climatic and seasonal factors [12]. From the previous studies, caryophyllene oxide was found as the most abundant essential oil component of *C. pelia*, *C. thessala* subsp. *drakiensis* and *C. zuccariniana* [13], *C. chrysantha* [14], *C. euxina* [15], *C. helenioides* [16], *C. amanicola*, *C. consanguinea* and *C. ptosimopappa* [17], *C. iberica* and *C. virgata* [18], *C. aucheri* [19] and *C. athoa* [20]. Furthermore, germacrene D was identified as a primary essential oil ingredient of *C. pseudoscabiosa* subsp. *pseudoscabiosa* and *C. hadimensis* [21], *C. kotschyi var. kotschyi* and *C. kotschyi var. decumbens* [22], *C. cineraria* subsp*. umbrosa* [23]. Meanwhile, hexadeconic acid is one of the significant compounds within the essential oil of *C. aggregata* subsp. *aggregata*, *C. balsamita* and *C. behen* [24], *C. stenolepis* [25], *C. solsitialis* [26] and *C. kilea* [27]. Notably, *C. iconiensis* contains a more unusual quantity of undec-1-ene (84.3%) [28]. Moreover, unusual compounds (arachidic acid, α-selinene and octanol) are recognized as active components in several *Centaurea* EOs [29,30]. Spathulenol is a volatile constituent of *C. aphrodisea* [20], *C. euxina* [21], *C. grisebachii* [31] and *C. lycopifolia* [30]. Monoterpenes are less than 10 % and sometimes rare or absent; among them, *α*- and *β*-pinenes, myrcene, α-phellandrene, *p*-cymene, *α*-copaene and limonene were the most constantly reported constituents [32,33].

To the best of our knowledge, this is the first report of EOs of *C. glomerata, C. scoparia* and *C. lipii* using hydrodistillation (HD) and microwave-assisted extraction (MAE). There are also few reports of *C. calcitrapa* and *C. alexandrina* EOs extracted by HD. As a result, a comparative study on the essential oil composition of these five wild Egyptian plants obtained by conventional techniques (HD) and innovated green technology (MAE) will be presented here. HD is frequently the method of choice for EO extraction even though it is time-consuming and can result in thermal degradation and/or hydrolysis for heat-sensitive volatiles [34].

Microwave-assisted extraction (MAE), in contrast, is an applicable method to recapture a wide array of compounds and secondary metabolites from plants compared with traditional reflux extraction methods [35]. MAE has a shorter extraction time and a higher selectivity, yield, and quality of EOs [36]. The current study aimed to (i) characterize the chemical constituents of the EOs of five *Centaurea* plants (i.e., *C. scoparia*, *C. calcitrapa*, *C. glomerata*, *C. lipii* and *C. alexandrina*) extracted by either hydro-distillation or microwave-assisted extraction techniques; (ii) apply principal component analysis (PCA) and agglomerative hierarchical clustering (AHC) to compare chemical profiles of the extracted EOs; and (iii) assay the extracted EOs for antimicrobial activity against *Staphylococcus aureus*, *Escherichia coli*, *Aspergillus niger*, and (IV) Gyrase B ATP-binding inhibition via in silico binding analysis.

## 2. Results and Discussion

### 2.1. EOs Chemical Profiles of *Centaurea* Species

EOs from five *Centaurea* species were extracted via HD and MAE. The yield of the extracted EOs by HD were found to be 0.03, 0.054, 0.043, 0.055 and 0.037% *v*/*w*, whereas by MAE they were 0.023, 0.031, 0.037, 0.044, and 0.024% *v*/*w* from *C. scoparia*, *C. calcitrapa*, *C. glomerata*, *C. lipii* and *C. alexandrina*, respectively. Table 1 summarized the compounds, retention time (Rt), Kovat’s index (KI), and percentage of each metabolite. EO yield variations are attributed to differences in the extraction method. Differences in environmental conditions may contribute to differences in EOs between the plant species [37].

**Figure 1:**
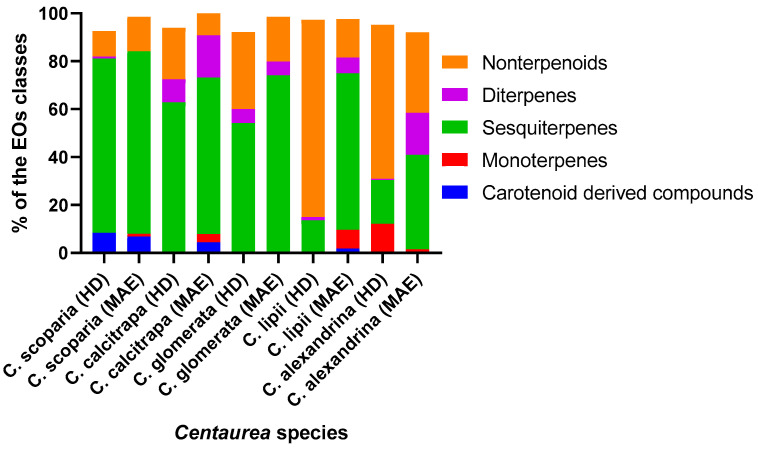
% of the EO classes of characterized compounds of the five *Centaurea* plants.

Terpenoids are the main components in EOs from *C. scoparia* via HD and MAE (73.47 and 76.06%, respectively); in addition, EOs contain nonterpenoids (10.64 and 14.46%) as well as carotenoid-derived compounds (8.41 and 6.86%), respectively (Figure 1). Among the identified terpenes, sesquiterpenes comprised the major component with a concentration of (72.68 and 76.06%), while diterpenes were detected in trace amounts (0.79 and 0%) and monoterpenes were not detected.

Two EOs derived from *C. calcitrapa* revealed the abundance of terpenoids with concentrations of 72.34 and 82.87% alongside with nonterpenoids (21.65 and 9.23%) (Figure 1). An amount of 4.48% of EO extracted by MW were characterized as carotenoid-derived compounds, while there was a complete absence of these compounds in HD-EO (Figure 1).

The EOs from *C. glomerata* displayed a preponderance of terpenoids (60.03 and 79.75%) as well as a high concentration of nonterpenoids (32.07 and 18.68%) (Figure 1). The data analysis exhibited a total absence of carotenoid-derived compounds in both methods, which agrees with a previous EO extraction of chemical components from *C. glomerata* [38].

The chemical profile of the HD-EO from *C. lipii* revealed a majority of nonterpenoids (82.37%) and a low concentration of terpenoids (14.96%) with complete absence of carotenoids. In contrast, extracted EO by MW terpenoids were 79.56%, comprising the main constituents, while a low concentration of nonterpenoids (16.11%) and traces of carotenoid-derived compounds (1.87%) was detected (Figure 1).

The obtained HD-EO of *C. alexandrina* was characterized by the abundance of nonterpenoids (64.25%), a significant concentration of terpenes (30.17%) and traces of carotenoid-derived compounds (0.46%). On the other hand, the terpenes were characterized as the main components of EO extracted via MW with concentration of 58.17% including mono- (1.24%), sesqui- (39.31%) and di- (7.63%) terpenes with the presence of a high concentration of nonterpenoids (33.53%) and an absence of carotenoid-derived compounds (Figure 1).

Collectively, sesquiterpenes were characterized as the main constituents in both extracted Eos of *C. scoparia*, with concentrations of 72.68 (HD) and 76.06% (MAE). Among the identified sesquiterpenes, caryophyllene oxide, (19.50 and 19.15%), spathulenol (12.96 and 11.98%), *trans*-caryophyllene (11.33 and 10.58%), torreyol (4.84 and 4.18%) and 6,10,14-trimethylpentadecan-2-one (4.45 and 5.03%) represented the main components. Similar to *C. scoparia*, sesquiterpenes were the main constituents of EOs of *C. calcitrapa*. Spathulenol (12.92 and 9.05%), caryophyllene oxide (12.38 and 12.14%), alloaromadendrene oxide-2 (7.92 and 9.14%), *α*-costol (5.88 and 4.39%) and 6, 10, 14-trimethyl pentadecan-2-one (3.49 and 8.56%) were characterized as the main sesquiterpenoids. These result were in accordance with the previous data on EOs of *Centaurea* plants such as *C. chrysantha* [14], *C. cheirolepidoides* [28], *C. consanguinea* [17] and *C. deflexa* [28].

Sesquiterpenes (54.24 and 74.07%) and diterpenes (5.79 and 5.68%) represented the overall categories of terpenoids in both EOs derived from *C. glomerata*. The disappearance and/or minority of mononterpenes in EOs of *C. scoparia* and *C. Calcitrapa*, as well as their total absence in the EOs for *C. glomerata*, is in agreement with the described analysis of EOs of different species of *Centaurea* [16,27].

The above analysis of EOs of *C. scoparia* and *C. calcitrapa*, as well as the reported data of EOs of different *Centaurea* ecoplants like *C. chrysantha* [14], *C. cheirolepidoides* [28], *C. consanguinea* [17] and *C. deflexa* [28], revealed that sesquiterpenes were found as the main constituents of EOs of *C. glomerata*. The compounds, spathulenol (3.70 and 18.77%), alloaromadendrene oxide-2 (11.52 and 15.18%), 6, 10, 14-trimethyl pentadecan-2-one (11.23 and 8.23%), *α*-costol (3.51 and 5.61%), guaiol (4.16 and 0%) and *α*-eudesmol (0 and 4.34%), represented the main sesquiterpenoid compound.

The abundance of hydrocarbons in hydro-distilled EO from *C. lipii*, especially that of fatty acids, is in agreement with the two *Centaurea* plants, *C. calcitrapa* and *C. spaerocephala*. Furthermore, previous studies of C. *pannonica* essential oil extracted by HD revealed that oil was rich in fatty acids (43.7%), with 9-octadecenoic acid (34.0%) and (*Z*,*Z*)-9,12-octadecadienoic acid (8.6%) as the major compounds [39]. However, 79.56% from total mass of EO extracted via MW were characterized as terpenoids, including 65.14% of sesquiterpenes in addition to 6.59% diterpenes and 7.83% monoterpenes. These findings were totally in agreement with the observation of EOs of the above three *Centaurea* plants (*C. scoparaia*, *C. calcitrapa*, *C. glomerata*), as well as the previous described data of EOs of the other *Centaurea* ecoplants [14,17,28]. Alloaromadendrene oxide-2 (31.55%) was found to be the main sesquiterpenoid of EO extracted via MW, as well as spathulenol (8.14%), 6,10,14-trimethylpentadecan-2-one (8.34%) and torreyol (7.11%). Most of these major compounds were characterized from all EOs of the above plants in addition to others such as *C. iberica*, *C. virgate* [18], *C. kilaea* [27] and *C. helenioides* [16].

Sesquiterpenoids (39.30%) were identified as the main class of EO extracted from *C. alexandrina* via MW in addition to a high concentration of diterpenes (17.63%) and minors of monoterpenes (1.56%). Caryophyllene oxide, (17.01%), 6, 10, 14-trimethyl pentadecan-2-one (14.23%) and aromadendrene oxide-1 (3.12%) were proven to be the major characterized sesquiterpenes. Thunbergol (9.05%), 13-*epi*-manool (6.53%) and phytol (1.13) were assigned as the main diterpene constituents. Only two monterpenes, 1, 8-cineole and boronal, were assigned as overall identified monoterpenes. All these findings were in complete agreement with the above four *Centaurea* plants in addition to the documented data of others such as *C. iberica*, *C. virgate* [18], *C. kilaea* [27] and *C. helenioides* [16].

Monoterpenes, basically constructed from an isoprene unit, were recognized as the main components in most of the EOs derived from the plant kingdom [40]. However, several *Centaurea* species were described to include traces and/or absence of monoterpenes such as *C. appendicigera*, *C. helenioides* [16] and *C. kilaea* [27]. These data agree with our results of *C. scoparia*. At the same time, diterpenes are described as rare components in EOs with some exceptions, such as *Lactuca serriola* [41] and the Indian leaves of *Araucaria heterophylla* [42]. Our results with *C. scoparia* are also in line with diterpenes found in trace amounts in EO extracted by hydro-distillation, with one compound (phytol, 0.79%), while the was a complete absence in EOs extracted via microwave.

Similar to the present data of EOs of *C. scoparia* and the reported EO constituents of the EOs of *C. appendicigera*, *C. helenioides* [16] and *C. kilaea* [27], the non-existence of monoterpenes were recorded in EOs of *C. calcitrapa*. In contrast with the data of EOs of *C. scoparia* and most investigated *Centaurea* species, this revealed the presence of substantial concentrations of diterpenoids with abundance of phytol in EOs of *C. scoparia* (7.90 and 16.89%, respectively). The abundance of phytol was already reported for *Centaurea* species such as *C. aggregata* ssp. *aggregate*, *C. behen* [24], *C. stenolepis* [25] and others. In complete agreement with the above two *Centaurea* plants and previous published data of others [16,27], the nonexistence of monoterpenes were observed from the EOs of *C. glomerata*. On the other side, low concentrations of diterpenes (5.79 and 5.68%) were identified in the two EOs of this plant with only one identified compound, phytol, that is already characterized from numerous *Centaurea* plants [24,27].

Like the above three plant (*C. glomerata, C. scoparia, C. calcitrapa*) and others [16,27], the presence of traces of monoterpenes were noticed only in extracted EO from *C. lipii*, by MAE, in a concentration of 7.83% with *d*-isothujone (3.12%) as the main component. From the extracted EO by HD, only two diterpenoids, dehydroabietane and *trans*-geranyl geraniol (0.70%, each), were identified from EO extracted via hydrodistillation, while only one compound, phytol (6.59%), was characterized from extracted EO by MW.

The nonterpenoid compounds of EOs of *C. scoparia* were categorized with considerable concentrations in the two EO samples (10.64% and 14.46%). Methyl palmitate was the main nonterpenoidal compound with concentrations of 2.71% and 6.69%. Both EO samples of *C. scoparia* were found to contain carotenoid-derived compounds with respective concentrations of 8.41% and 6.86%. Theaspirane A (3.75 and 3.16%) and theaspirane B (2.00 and 1.91%) were characterized as the main compounds. The high concentration of the nonterpenoids in EOs of *C. scoparia* was in accordance with the previous described EOs of the *Centaurea* species, such as *C. kilaea* [27], *C. amanicola* [17], *C. armena* [43], *C. cadmea* and *C. calolepis* [44].

The nonterpenoids constitute 21.65 and 9.23% in *C*. *calcitrapa* EOs in both extraction methods, but only MAE of C. *calcitrapa* showed carotenoid-derived compounds with a concentration of 4.48%. From the overall identified nonterpenoids, arachidic acid (11.05%), 7, 10-octadecadienoic acid, methyl ester (3.65%) and paeonol (3.35%) represent the main compounds of HD-EO. These results were in complete uniformity with published data of EO of *C. balsamita* in which arachidic acid (25.3 %) was considered as a major component [29]. Moreover, boronal (3.41%) and *Z*-7-hexadecenal (1.82%) are the major compounds of extracted EO of *C. calcitrapa* by MW. The overall mass (4.48%) of EOs derived via MAE were characterized as carotenoids including (*E*)-*β*-damascenone (1.52%) and *trans*-α-ionone (1.49%) as the main constituents.

The two extracted EOs from *C. glomerata* nonterpenoids were characterized by high concentrations (32.07 and 18.68%), in which *n*-tricosane (10.51 and 1.64%) and phenylethyl stearate (4.41 and 3.35%) were found as major components.

The nonterpenoids (82.37%) represent the main components of the extracted EOs of *C. lipii* via hydrodistillation, in which *n*-heneicosane (61.07%) and *n*-eicosane (12.68%) were found as the main constituents. This result is completely in contrast with the above EOs of the three *Centaurea* plants, *C. scoparia*, *C. calcitrapa* and *C. glomerata*, and also with the extracted EO by MAE from the same plant. These results strongly elucidate the effect of the extraction method on the chemical composition of EOs. In the other side, 16.11% from the total mass of EOs derived via MAE were characterized by the hydrocarbon *n*-heneicosane (6.71%) and phenylethyl stearate (2.67%), which constitute the main entities.

The nonterpenoids of the extracted EO from *C. alexandrina* via hydro-distillation were assigned as (*Z*)-9-octadecenamide (19.13%), methyl arachidonate (14.25%), arachidic acid (9.81%) and phenylethyl stearate (6.82%). These results were in complete uniformity with the published data of the EOs of *C. lycopifolia*, in which arachidic acid (5.0 %) was considered as one of the major components [30]. In EOs extracted by MAE from *C. alexandrina*, the nonterpenoids represented by high concentrations with an abundance of methyl arachidonate (12.52%), (*Z*)-9-octadecenamide (3.92%) and phenylethyl stearate (3.70%). These results were in complete agreement with the results of the EOs of *C. lipii*, especially that extracted via hydrodistillation and also with the published data of the EOs of *C. calcitrapa* and *C. spaerocephala*. This significant un-symmetry strongly supports the theory of the effects of the extraction techniques on the EOs chemical components.

### 2.2. Unsupervised PCA and HCA Data Analyses

**Figure 2:**
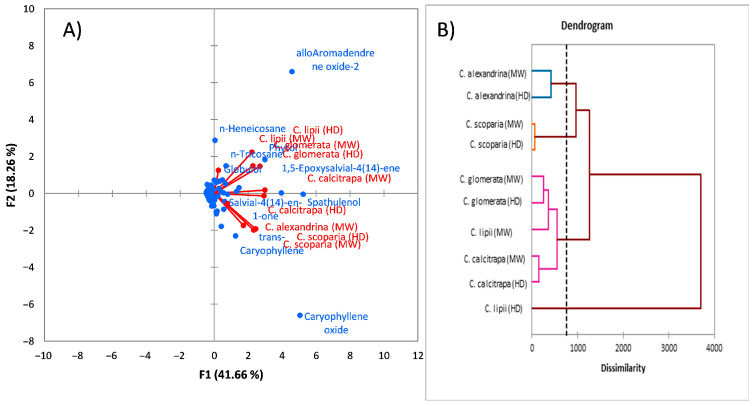
(**A**) Principal component analysis (PCA); (**B**) Agglomerative hierarchical clustering (AHC) of the five *Centaurea* species, *C. scoparia*, *C. calcitrapa*, *C. glomerata*, *C. lipii* and *C. alexandrina*, based on the major chemical compounds of the extracted EOs by hydrodistillation and microwave.

The correlation between the five *Centaurea* species was established based upon the main compounds of the EOs via PCA and AHC (Figure 2A,B). The PCA exhibited an explanation of 35.92 and 22.08% of the overall variance in the horizontal and vertical axes.

The PCA and AHC data revealed a strong correlation between the five plants. Firstly, *C. calcitrapa* exhibited strong correlation with *C. scoparia* and moderate correlation with *C. alexandrina*. Similarly, *C. scoparia* and *C. calcitrapa* exhibited a weak correlation with *C. alexandrina*. Furthermore, *C. glomerata* and *C. lipii* exhibited strong correlations, especially via the EO derived by microwave. The significant variations between the five plants might be attributed to the microclimatic and environmental condition variations [41,45].

*C. scoparia* and *C. calcitrapa* exhibited a strong correlation depending on the main components in which spathulenol, caryophyllene oxide, *trans*-caryophyllene and 6, 10, 14-trimethyl pentadecan-2-one were found as the main constituents. Caryophyllene oxide and spathulenol were characterized as major components in the EOs of the three plants *C. scoparia*, *C. calcitrapa* and *C. alexandrina*. Otherwise, the correlations of the two plants *C. glomerata* and *C. lipii* were deduced via the main compounds of the EOs of the plants, including spathulenol, alloaromadendrene oxide-2,6,10,14-trimethyl pentadecan-2-one and phytol. The correlations of the five plants were also observed via the main compounds of their EOs, such as spathulenol, 6,10,14-trimethyl pentadecan-2-one and phytol.

In conclusion, the results of chemometrics analysis deduced that there are significant variations between the five *Centaurea* species in EO composition depending upon the variations of the plant species and/or extraction method. Moreover, the significant variations might be attributed to the variations of the plant species more than the variations of the extraction method. Therefore, our results confirmed the effects of variations in plant species and extraction methods on the quantity and quality of EO composition.

### 2.3. Antimicrobial Activity

The microdilution assay was used to determine MIC values for EOs of the five *Centaurea* species against *S. aureus*, *E. coli* and *A. niger*. Strikingly, MAE-EOs of *C. glomerata*, *C. lipii* and *C. calcitrapa* showed a potent antibacterial activity against *S. aureus*, with MIC 31.25 µg/mL (Table 2). Our results suggest that MAE is the method of choice for extraction of *Centaurea* EOs for the purpose of antimicrobial activity.

The major constituents of the EOs may play a basic role as antimicrobial agents, whether as a singular role or synergistically with the other compounds. For example, alloaromadendrene oxides, as major components, were described to have an antimicrobial role in EOs in several plants such as *Lippia alb* [46], *Aloysia citriodora* [47], *Curcuma aeruginosa* [48] and others. According to the literature, spathulenol was found to be effective against several bacterial and fungal pathogens [49]; 6, 10, 14-Trimethyl pentadecan-2-one is one of the most common compounds from EOs of plants. This compound was described to have an important role in the antimicrobial activities of EOs derived from the flowers of several plants such as *Citrus aurantium* L [50]. Based on these studies, these major compounds might have a principal role in the antimicrobial activities of EOs. In addition to these main components, there are other major and minor compounds that are reported to have antimicrobial potentialities. Finally, the synergistic effects of the main and minor constituents of the EOs might be a possible pathway into the bioactivity of the oils.

### 2.4. Molecular Docking Inhibitory Effect of EOs Major Metabolites on ATP- Binding Pocket of Gyrase B Enzyme and N-Myristoyltransferase

**Figure 3:**
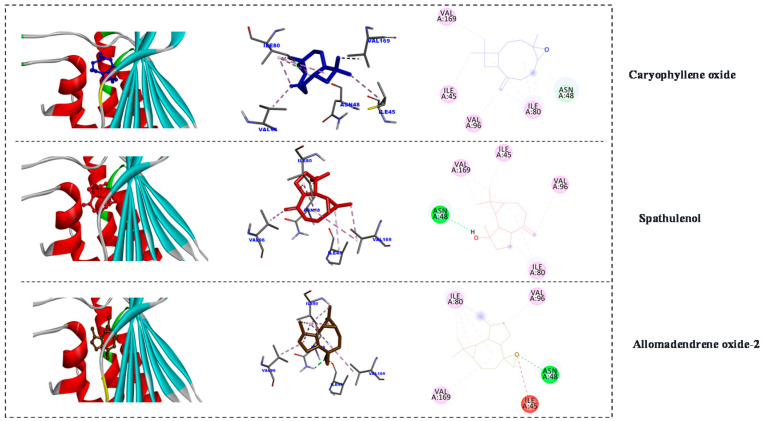
2D and 3D representations of the predicted binding modes, as well as the docking scores, of EO major compounds inside the active site of 4GEE (antibacterial).

The advantages of computer-aided drug discovery via structural studying against targeted enzymes as well as drug repurposing have led them to become an essential program for major pharmaceutical companies, owing to the speed and lower costs of their processes. Recently, DNA gyrases have become an attractive target for anticancer and antibacterial research as they are essential enzymes for cell survival in prokaryotes. Today, searching for new inhibitors of the ATP-binding pocket of Gyrase B enzyme (PDB code: 4GEE) is attracting the attention of pharmaceutical industries [51]. Alloaromadendrene oxide-2 and spathulenol showed the best binding affinity (−6.98 and −6.88 kcal mol^−1^), while caryophyllene oxide showed slightly less binding affinity (−6.35 kcal mol^−1^). Additionally, the inhibition constant (pKi) for the tested metabolites was as follows: alloaromadendrene oxide-2 (7.63 µM), spathulenol (9.03 µM) and caryophyllene oxide (22.34 µM). The positive control (Thiophenicol) binding affinity was −6.97 kcal mol^−1^ and the inhibition constant (pKi) was 7.73 µM (Figure 3).

A specific lipidic modification for the -*N*-terminal glycine residue mainly sign to *N*-myristoylation protein in many viral and eukaryotic proteins [52].

**Figure 4:**
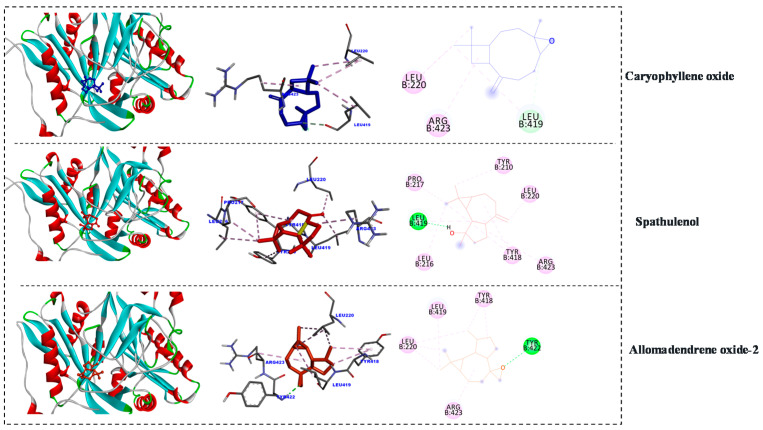
2D and 3D representations of the predicted binding modes, as well as the docking scores, of EO major compounds inside the active site of 1IYK (antifungal).

The binding score of alloaromadendrene oxide-2 (−7.50 kcal mol^−1^) and spathulenol (−7.71 kcal mol^−1^) with *N*-myristoyltransferase (PDB Code: 1IYK) and moderate fitting was observed in the case of caryophyllene oxide (−6.71 kcal mol^−1^). The inhibition constant (pKi) for tested metabolites were 3.18, 2.22, and 12.11 µM, respectively). The positive control (Thiophenicol) binding affinity was −6.12 kcal mol^−1^ and the inhibition constant (pKi) was 32.53 µM (Figure 4).

## 3. Materials and Methods

### 3.1. Plant Material

The aerial parts of *C. scoparia*, *C. calcitrapa*, *C. glomerata*, *C. lipii* and *C. alexandrina* were collected during the flowering stage in April 2017 from Saint-Catherine-Southern Sinai Governorate, Ismalia City-Ismalia Governorate, Rashid centre-Beheira Governorate, Mediterranean coastal belt at Alexandria City and Borg El-Arab City-Alexandria Governorate, Egypt respectively. All *Centaurea* species under study were deposited in National Research Centre herbarium and were identified by the taxonomist Dr. I. El-Garf, (Professor of Botany, Faculty of Science, Cairo University, Cairo, Egypt) with and voucher specimens as the following: *C. scoparia* no. M/2278, *C. calcitrapa* no. M/2279, *C. glomerata* no. M/2280, *C. lipii* no. M/2281 and *C. alexandrina* no. M/2282.

### 3.2. Extraction of EO by Hydrodistillation

The dried aerial parts (250 g) of investigated *Centaurea* species were extracted for 3 h over a Clevenger-type apparatus using our previous protocol [53]. The EO samples kept were stored in sealed air-tight glass vials at 4 °C for analyses.

### 3.3. Microwave-Assisted Extraction of EOs

A microwave apparatus working at 2450 MHz and 1600 W maximum power (CEM Corporation, Matthews, NC, USA) was used to examine the plant species (250 g) under study using our previously reported protocol [53]. Extraction parameters: power, time and temperature were 800 W, 60 min and 100 °C respectively. All extracted EOs were dried using anhydrous sodium sulfate and kept directly in 4 °C till analysis.

### 3.4. GC–MS Analysis

Gas Chromatography-Mass Spectrometry (GC-MS) (THERMO Scientific Corp., Waltham, MA, USA), connected with a thermo mass spectrometer detector (ISQ Single Quadrupole Mass Spectrometer; Model ISQ spectrometer, electron ionization (EI) at 70 eV, *m*/*z* 40–450 a spectral range of), was used for plants EO analyses. A TR-5 MS (30 m × 0.32 mm i.d., 0.25 μm) column and helium as carrier gas (flow rate (one mL/min; split ratio (1:10); temperature program (60°C for one min; rising at 4.0 °C/min to 240°C and held for one min) were used and both the injector and detector were held at 210°C. One μL of the mixtures diluted by *n*-hexane (1:10, *v*/*v*) were injected.

### 3.5. Identification of EO Constituents

The EO’s main constituents were recognized via AMDIS software (www.amdis.net), according to its retention indices (relative to *n*-alkanes C_8_–C_22_), mass spectrum corresponding to authentic standards, Wiley spectral library collection and NIST library databases as well.

### 3.6. MS Data Processing for Multivariate Analysis: PCA and HCA

The data of the major compounds of extracted EOs of the five samples (*C. scoparia*, *C. calcitrapa*, *C. glomerata*, *C. lipii* and *C. alexandrina*) were subjected to an agglomerative hierarchical cluster (AHC) using XLSTAT statistical computer software package (version 2018, Addinsoft, New York, NY, USA, www.xlstat.com (accessed on 29 January 2021)). Moreover, the matrix of data was also submitted to correlation by a principal component analysis (PCA) to identify whether a significant correlation exists between different samples using XLSTAT also.

### 3.7. Antimicrobial Activity Assay

#### 3.7.1. Microorganisms

The microbial strains were provided by the culture collection of Microbial and Natural Products Chemistry Department, National Research Centre (NRC), Cairo, Egypt and maintained according to Elbatal et al., 2019. The microbial suspension was adjusted to 0.5 McFarland solution [54].

#### 3.7.2. Determination of Minimum Inhibitory Concentrations

The MIC values were determined by the broth microdilution assay (NCCLS, 2008) with slight modification [55]. The assay was carried out in nutrient broth medium for bacteria and potato dextrose broth medium for fungus. The assay was performed according to the reported procedure [56], with slight modifications. Briefly, 3 µL of the essential oil of concentration 1 mg/mL DMSO were prepared as an initial concentration in the first column of the sterile polystyrene 96 well plates. Then 197 µL of the tested microbial suspension adjusted to 5 × 10^5^ CFU/mL was added. Serial dilutions were done by addition of 100 µL of the first column to the second one and so on. The final volume was adjusted to 200 µL on each well by addition of the microbial suspension to get final concentrations of tested compounds from 31.25 to 1000 µg. Negative control was made by adding an equal volume of DMSO instead of tested extracts. Blank control was prepared using broth medium. The plates were incubated in sterile conditions at a suitable temperature for the microbial growth. The turbidity of the culture media was taken as an indicator for microbial growth. The MIC value was taken as the lowest concentration of the test agent that caused complete inhibition (100%) of microbial growth [57]. Thiophenicol (Thiamphenicol, Sanofi-Aventis, France) and triflucan (Fluconazole, Egyptian International Pharmaceutical Industries Company (EIPICO), Naser City, Cairo, Egypt) were used as antibacterial and antifungal positive control drugs, respectively.

### 3.8. Molecular Docking

The chemical structures of EO Major metabolites, caryophyllene oxide, spathulenol, and allomadendrene oxide-2, were downloaded as SDF files from the PubChem database (https://pubchem.ncbi.nlm.nih.gov/ (accessed on 29 January 2021)), followed by converting these files to PDB format via the free software Avogadro (https://avogadro.cc/ (accessed on 29 January 2021)). The protein crystal structures for 4GEE (antimicrobial) and 1iyk (anti-fungal) were downloaded from the protein databank (https://www.rcsb.org/ (accessed on 29 January 2021)) using our previously published molecular docking protocol [58].

## 4. Conclusions

In the present study, GC/MS analysis was used to investigate the chemical composition of five Egyptian *Centaurea* species of EOs obtained by HD and MAE techniques, and the impact of an extraction method on the composition of metabolites of each EO was further assessed. The qualitative and quantitative variations in the composition of essential oils prepared by MAE and HD is probably due to climatic and environmental factors as well as to the possible degradation of products by hydrolysis, oxidation and trans-esterification, due to prolonged extraction time of HD when compared to MAE. These results could be useful in designing the best extraction method. Interestingly, it is the first report on the analysis of essential oils of *C. lippi*, *C. glomerata* and *C. scoparia*. Sesquiterpenes constitute the major classes in MAE- and HD-EOs of *C. scoparia, C. calcitrapa* and *C. glomerata*. In general, the amount of total terpenoids in MAE-EOs were higher than the HD-EOs. In contrast, the percentage of nonterpenoids for all studied species was higher in the HD-EOs than the MAE-EOs except for *C. scoparia*. The possible reason for this contradictory result is that the content of nonterpenoids of the oil was dependent on the species instead of the extraction method. The EOs of *C. lipii*, *C. glomerata* and *C. calcitrapa*, isolated by MAE in this study, exhibited significant inhibitory responses against the *S. aureus*, likely due to their enrichment in sesquiterpene compounds. In silico exploration, alloaromadendrene oxide-2 and spathulenol exhibited a significant inhibition of the ATP- binding pocket of the Gyrase B enzyme. These phytoconstituents could serve as potential candidates for the discovery of antibacterial and antifungal drugs, however their therapeutic potential is yet to be validated using in vitro and in vivo studies.

## Figures and Tables

**Table 1:** EOs constituents of *C. scoparia*, *C. calcitrapa*, *C. glomerata*, *C. lipii* and *C. alexandrina* extracted by HD and MAE.

No	RT	KI	Compound Name	*C. scoparia*		*C. calcitrapa*		*C. glomerata*		*C. lipii*		*C. alexandrina*		*Identification*
				HD	MAE	HD	MAE	HD	MAE	HD	MAE	HD	MAE	
**Monoterpenes**														
1	4.18	933	2,6,6-trimethylbicyclo[3.1.1]hept-2-ene (*α*-Pinene)	-	-	-	-	-	-	-	-	0.65	-	a, b & c
2	6.77	1031	1-methyl-4-prop-1-en-2-ylcyclohexene (Limonene)	-	-	-	-	-	-	-	-	0.82	-	a, b & c
3	6.98	1039	1,3,3-trimethyl-2-oxabicyclo[2.2.2]octane (1,8-Cineole)	-	-	-	-	-	-	-	1.56	**9.24**	**1.24**	a & b
4	9.36	1063	(2*S*,5*R*)-2-methyl-5-propan-2-ylbicyclo[3.1.0]hexan-2-ol (*trans*-Sabinene hydrate)	-	-	-	-	-	-	-	-	0.43	-	a & b
5	9.79	1098	(1*S*,4*S*,5*R*)-4-methyl-1-propan-2-ylbicyclo[3.1.0]hexan-3-one (Isothujone)	-	-	-	-	-	-	-	**3.12**	-	-	a & b
6	10.23	1100	(1*S*,4*R*,5*R*)-4-methyl-1-propan-2-ylbicyclo[3.1.0]hexan-3-one (α-Thujone)	-	-	-	-	-	-	-	0.70	-	-	a & b
7	11.44	1139	1,7,7-trimethylbicyclo[2.2.1]heptan-2-one (Camphor)	-	-	-	-	-	-	-	0.57	0.30	-	a, b & c
8	12.42	1158	1,7,7-trimethylbicyclo[2.2.1]heptan-2-ol (Borneol)	-	-	-	-	-	-	-	1.23	-	-	a, b & c
9	13.39	1178	2,6,6-Trimethylcyclohexa-1,3-diene-1-carbaldehyde (Safranal)	-	-	-	-	-	-	-	-	0.23	-	a & b
10	16.03	1167	(2*R*,5*S*)-5-methyl-2-prop-1-en-2-ylcyclohexan-1-one (trans-Isopulegone)	-	-	-	-	-	-	-	0.65	-	-	a & b
11	25.39	1584	(*E*)-2-Methyl-4-(2,6,6-trimethylcyclohexen-1-yl)but-2-enal (Boronal)	-	1.18	-	**3.41**	-	-	-	-	-	0.32	a & b
**Sesquiterpenes**														
12	20.17	1405	3,3,7-trimethyl-8-methylidenetricyclo[5.4.0.02,9]undecane (Longifolene)	2.00	1.03	-	-	-	-	-	-	-	-	a & b
13	22.27	1418	(1*R*,4*E*,9*S*)-4,11,11-trimethyl-8-methylidenebicyclo[7.2.0]undec-4-ene (*trans*-Caryophyllene)	0.87	0.67	0.73	-	-	-	-	-	0.26	-	a & b
14	22.5	1419	2,6,6,8-tetramethyltricyclo[5.3.1.01,5]undec-8-ene (*α*-Cedrene)	0.65	-	-	-	-	-	-	-	-	-	a & b
15	25.21	1428	(1*R*,4*E*,9*S*)-4,11,11-trimethyl-8-methylidenebicyclo[7.2.0]undec-4-ene (*trans*-Caryophyllene)	**11.33**	**10.58**	-	-	3.40	-	-	-	-	-	a & b
16	25.63	1485	(3*R*,8a*S*)-5,8a-dimethyl-3-prop-1-en-2-yl-2,3,4,4a,7,8-hexahydro-1H-naphthalene (*α*-Selinene)	-	0.58	-	-	-	-	-	-	-	1.59	a & b
17	26.37	1493	(1a*R*,7*R*,7a*S*,7b*R*)-1,1,4,7-tetramethyl-1a,2,3,5,6,7,7a,7b-octahydrocyclopropa[e]azulene (Ledene)	0.71	-	-	-	-	-	-	-	-	-	a & b
18	26.77	1497	(1*S*,4a*S*,8a*R*)-4,7-dimethyl-1-propan-2-yl-1,2,4a,5,6,8a-hexahydronaphthalene (*α*-Muurolene)	-	-	-	0.78	-	-	-	-	-	-	a & b
19	27.01	1514	(3*R*,4a*R*,5*S*)-4a,5-dimethyl-3-prop-1-en-2-yl-2,3,4,5,6,7-hexahydro-1H-naphthalene (Eremophilene)	-	-	-	-	-	1.38	-	-	-	-	a & b
20	27.16	1515	(4a*R*)-3,5,5,9-tetramethyl-1,2,4a,6,7,8-hexahydrobenzo[7]annulene (*β*-Himachalene)	-	-	-	-	-	-	-	-	-	0.33	a & b
21	28.00	1527	(1*S*,4*S*,4a*S*,6*R*,8a*R*)-1,6-dimethyl-4-propan-2-yl-1,2,3,4,4a,5,6,8a-octahydronaphthalene (*α*-Cadinene)	-	0.48	-	1.50	-	0.72	-	0.64	-	-	a & b
22	28.03	1539	(4a*R*,8a*R*)-5,8a-dimethyl-3-propan-2-ylidene-1,2,4,4a,7,8-hexahydronaphthalene (Eudesma-3,7(11)-diene)	-	-	1.69	-	1.13	1.44	-	-	-	-	a & b
23	28.27	1548	(6*E*)-3,7,11-trimethyldodeca-1,6,10-trien-3-ol (Nerolidol)	-	-	-	1.01	-	-	-	-	-	-	a & b
24	28.39	1554	2-methyl-8-methylidene-5-propan-2-yl-11-oxatricyclo[5.3.1.02,6]undecane (1,5-Epoxysalvial-4(14)-ene)	0.96	-	1.59	2.09	1.39	4.94	-	1.77	-	-	a & b
25	28.8	1563	(1a*R*,4a*R*,7*S*,7a*R*,7b*R*)-1,1,7-trimethyl-4-methylidene-1a,2,3,4a,5,6,7a,7b-octahydrocyclopropa[h]azulen-7-ol (Spathulenol)	**12.96**	**11.98**	**12.92**	**9.05**	**3.70**	**18.77**	1.71	**8.14**	**1.61**	0.39	a & b
26	29.14	1575	1,1,4,7-tetramethyl-2,3,4a,5,6,7,7a,7b-octahydro-1aH-cyclopropa[e]azulen-4-ol (Globulol)	0	0	2.19	1.85	1.08	3.72	-	1.52	-	-	a & b
27	29.39	1576	(1*S*,3a*R*,8a*S*)-3a-methyl-7-methylidene-1-propan-2-yl-2,3,5,6,8,8a-hexahydro-1*H*-azulen-4-one (Salvial-4(14)-en-1-one)	2.40	2.94	2.94	3.53	-	0.77	-	1.61	0.62	0.40	a & b
28	29.56	1579	2-[(3*S*,5*R*,8*S*)-3,8-dimethyl-1,2,3,4,5,6,7,8-octahydroazulen-5-yl]propan-2-ol (Guaiol)	0.84	0.75	-	-	-	1.10	-	0.65	-	-	a & b
29	30.41	1691	1,4a-dimethyl-7-propan-2-ylidene-3,4,5,6,8,8a-hexahydro-2H-naphthalen-1-ol (Juniper camphor)	1.56	1.39	2.46	2.23	0.97	2.09	1.15	0.75	-	-	a & b
30	30.74	1592	6,7,9,9-tetramethyl-2-oxatetracyclo[5.5.0.01,3.08,10]dodecane (Calarene epoxide)	-	-	-	-	2.07	-	-	-	-	-	a & b
31	30.89	1595	2-[(3*R*,5*S*,8*R*)-3,8-dimethyl-1,2,3,4,5,6,7,8-octahydroazulen-5-yl]propan-2-ol (α-Guaiol)	2.89	2.44	-	-	**4.16**	-	-	-	-	-	a & b
32	30.98	1597	[(2*E*)-3,7-dimethylocta-2,6-dienyl] 3-methylbutanoate (Geranyl isovalerate)	-	-	-	-	-	-	-	0.79	-	-	a & b
33	31.01	1600	(1a*R*,4*S*,4a*S*,7*R*,7a*S*,7b*S*)-1,1,4,7-tetramethyl-2,3,4a,5,6,7,7a,7b-octahydro-1aH-cyclopropa[e]azulen-4-ol (Viridiflorol)	-	-	-	0.71	-	-	-	-	-	-	a & b
34	31.10	1612	2,7,7,10-tetramethyl-3-oxatetracyclo[7.3.0.02,4.06,8]dodecane (Isoaromadendrene epoxide)	-	-	-	-	-	1.10	-	0.54	-	-	a & b
35	31.16	1618	2,2,6-trimethyl-10-methylidenetricyclo[5.3.1.01,6]undecan-9-ol (Longipinocarveol, *trans*)	-	-	-	-	2.07	-	-	-	-	-	a & b
36	31.39	1619	(2*Z*,8*E*)-3,7,7,10-tetramethylcycloundeca-2,8-dien-1-ol (Humulane-1,6-dien-3-ol)	-	-	-	-	-	-	-	-	0.60	0.66	a & b
37	31.58	1631	(6*S*,8*S*,9*R*,10*S*)-3,7,7,10-tetramethyl-2-oxatetracyclo[7.3.0.01,3.06,8]dodecane (Ledene oxide)	3.15	-	-	-	-	-	-	-	-	-	a & b
38	31.59	1633	3,7,7,10-tetramethyl-2-oxatetracyclo[7.3.0.01,3.06,8]dodecane (Ledene oxide (II))	-	2.78	-	-	-	-	-	-	-	-	a & b
39	31.62	1640	(1*S*,4*S*)-1,6-dimethyl-4-propan-2-yl-3,4,4a,7,8,8a-hexahydro-2H-naphthalen-1-ol (Cadinol)	-	-	0.92	1.05	-	-	-	-	-	-	a & b
40	31.84	1649	(1*R*,4*S*,4a*R*,8a*S*)-1,6-dimethyl-4-propan-2-yl-3,4,4a,7,8,8a-hexahydro-2H-naphthalen-1-ol (Torreyol)	**4.84**	**4.18**	-	-	3.34	-	-	**7.11**	0.80	0.83	a & b
41	31.88	1652	2-[(2*R*,4a*S*)-4a,8-dimethyl-2,3,4,5,6,7-hexahydro-1H-naphthalen-2-yl]propan-2-ol (Eudesmol)	-	-	-	-	-	**4.34**	-	-	-	-	a & b
42	31.89	1653	(1*S*,4*R*)-1,6-dimethyl-4-propan-2-yl-3,4,4a,7,8,8a-hexahydro-2H-naphthalen-1-ol (*α*-Cadinol)	-	-	**6.29**	**5.31**	-	-	-	-	-	-	a & b
43	31.93	1662	(1a*S*,4a*R*,7a*S*,7b*R*)-1,1,7-trimethylspiro[2,3,4a,5,6,7,7a,7b-octahydro-1aH-cyclopropa[e]azulene-4,2′-oxirane] (Aromadendrene oxide-1)	-	-	-	-	-	-	0.39	-	**3.41**	**3.12**	a & b
44	32.19	1678	(1a*R*,4*S*,4a*R*,7*R*,7a*S*,7b*S*)-1,1,7-trimethylspiro[2,3,4a,5,6,7,7a,7b-octahydro-1aH-cyclopropa[e]azulene-4,2′-oxirane] (Aromadendrene oxide-2)	-	-	**7.92**	**9.14**	**11.52**	**15.18**	**10.00**	**31.55**	-	-	a & b
45	32.20	1688	(1*S*,4a*S*)-1,4a-dimethyl-7-propan-2-ylidene-3,4,5,6,8,8a-hexahydro-2H-naphthalen-1-ol (Eudesm-7(11)-en-4-ol)	0.74	8.30	-	-	-	-	-	-	-	-	a & b
46	32.35	1693	(*Z*)-5-(2,6-dimethyl-6-bicyclo[3.1.1]hept-2-enyl)-2-methylpent-2-en-1-ol (Z-α-trans-Bergamotol)	-	-	-	-	-	-	-	-	-	0.74	a & b
47	32.47	1729	2-(*2R*,4a*R*,8a*R*)-4a,8-Dimethyl-1,2,3,4,4a,5,6,8a-octahydronaphthalen-2-yl)prop-2-en-1-ol (α-Costol)	2.83	2.35	**5.88**	**4.39**	**3.51**	**5.61**	-	-	-	-	a & b
48	32.61	1755	(4a*S*,7*R*)-1,4a-dimethyl-7-prop-1-en-2-yl-3,4,5,6,7,8-hexahydronaphthalen-2-one (α-Cyperone)	-	0.93	-	-	-	-	-	-	-	-	a & b
49	32.62	1763	6,6,8,9-tetramethyl-2-oxatetracyclo[6.4.0.01,3.05,7]dodecane (Aristolene epoxide)	-	-	-	-	-	0.97	-	1.15	-	-	a & b
50	33.06	1772	[(*Z*)-3-(3,7-dimethyl-2,4,5,6,7,7a-hexahydro-1H-inden-4-yl)-2-methylprop-2-enyl] acetate ((Z)-Valerenyl acetate)	-	-	1.42	1.11	1.08	3.12	-	1.52	-	-	a & b
51	33.27	1836	6,10,14-trimethylpentadecan-2-one	4.45	5.03	3.49	8.56	11.23	8.23	0.31	8.34	10.00	14.23	a & b
52	34.21	1915	(5*E*,9*E*)-6,10,14-trimethylpentadeca-5,9,13-trien-2-one (Farnesylacetone)	-	0.50	-	-	-	-	-	-	-	-	a & b
53	35.10	1929	7,9-ditert-butyl-1-oxaspiro[4.5]deca-6,9-diene-2,8-dione	-	-	-	-	-	0.59	-	-	-	-	a & b
54	35.16	1950	3-[(2*E*)-3,7-dimethylocta-2,6-dienyl]-2,6,6-trimethyl-3-[2,6,6-trimethyl-3-(2,6,6-trimethyl-3-bicyclo[3.1.1]hept-1-enyl)-3-bicyclo[3.1.1]hept-1-enyl]bicyclo[3.1.1]hept-1-ene (Geranylterpinene)	-	-	-	-	**3.59**	-	-	-	-	-	a & b
55	40.80	2013	(3*R*,4a*R*,6a*S*,10a*S*,10b*R*)-3-ethenyl-3,4a,7,7,10a-pentamethyl-2,5,6,6a,8,9,10,10b-octahydro-1H-benzo[f]chromene (Manoyl oxide)	-	-	-	0.78	-	-	-	-	-	-	a & b
**Diterpenes**														
56	43.63	1961	(3*S*)-5-[(1*S*,4a*S*,8a*S*)-5,5,8a-trimethyl-2-methylidene-3,4,4a,6,7,8-hexahydro-1H-naphthalen-1-yl]-3-methylpent-1-en-3-ol (13-*epi*-Manool)	-	-	-	-	-	-	-	-	-	**6.53**	a & b
57	44.87	2073	(2*E*,7*E*,11*E*)-1,7,11-trimethyl-4-propan-2-ylcyclotetradeca-2,7,11-trien-1-ol (Thunbergol)	-	-	1.62	0.75	-	-	-	-	-	**9.05**	a & b
58	47.95	2084	(4a*S*,10a*S*)-1,1,4a-trimethyl-7-propan-2-yl-2,3,4,9,10,10a-hexahydrophenanthrene (Dehydroabietane)	-	-	-	-	-	-	0.70	-	-	0.46	a & b
59	46.99	2114	(E,7*R*,11*R*)-3,7,11,15-tetramethylhexadec-2-en-1-ol (Phytol)	0.79	-	**7.90**	**16.89**	**5.79**	**5.68**	-	6.59	**0.52**	**1.13**	a & b
60	51.30	2201	(2*E*,6*E*,10*E*)-3,7,11,15-tetramethylhexadeca-2,6,10,14-tetraen-1-ol (Geranylgeraniol)	-	-	-	-	-	-	0.70	-	-	0.46	a & b
**Carotenoid derived compounds**														
61	17.18	1273	(2*S*,4a*R*,8a*R*)-2,5,5,8a-tetramethyl-3,4,4a,6-tetrahydro-2H-chromene (Dihydroedulan I)	-	2.00	-	-	-	-	-	-	-	-	a & b
62	17.34	1298	2,6,6,10-tetramethyl-1-oxaspiro[4.5]dec-9-ene (Theaspirane A)	**3.75**	**2.00**	-	0.70	-	-	-	-	-	-	a & b
63	18.07	1302	2,6,6,10-tetramethyl-1-oxaspiro[4.5]dec-9-ene (Theaspirane B)	**3.16**	**1.91**	-	0.77	-	-	-	-	-	-	a & b
64	20.69	1384	(*E*)-1-(2,6,6-trimethylcyclohexa-1,3-dien-1-yl)but-2-en-1-one(*β*-Damascenone)	0.68	-	-	1.52	-	-	-	-	0.46	-	a & b
65	21.99	1406	4-(2,6,6-trimethylcyclohex-2-en-1-yl)Butan-2-one (Dihydro-α-ionone)	0.82	0.95	-	-	-	-	-	-	-	-	a & b
66	25.03	1426	(*E*)-4-(2,6,6-trimethylcyclohexen-1-yl)but-3-en-2-one (*β* –Ionone)	-	-	-	1.49	-	-	-	1.87	-	-	a & b
**Nonterpenoids**														
67	9.90	1098	*n*-Nonanal	-	-	-	-	-	-	-	-	0.22	-	a & b
69	17.09	1179	Naphthalene	-	0.87	-	-	-	-	-	-	-	-	a & b
70	18.16	1292	Tridec-1-ene	-	-	-	-	-	-	-	1.29	-	-	a & b
71	19.45	1328	3,5,9,9-Tetramethyl-2-methylidenespiro[3.5]non-5-ene	-	0.65	-	-	-	-	-	-	-	-	a & b
72	20.6	1373	(2-ethyl-3-hydroxyhexyl) 2-Methylpropanoate	-	0.76	-	-	-	-	-	-	-	-	a & b
73	22.26	1380	(3-hydroxy-2,2,4-trimethylpentyl) 2-methylpropanoate (Texanol)	-	-	-	1.50	-	-	-	-	-	-	a & b
74	24.83	1438	1-(2-hydroxy-4-methoxyphenyl)ethanone (Paeonol)	-	-	**3.35**	-	-	0.58	-	1.05	-	-	a & b
75	24.85	1538	2,6,10-Trimethyltetradecane	-	-	-	-	-	-	0.39	-	-	-	a & b
76	27.94	1620	1-Decylsulfanyldecane (Decyl Sulfide)	1.54	1.37	-	-	-	-	-	-	-	-	a & b
77	32.24	1632	Tetradecanal	-	-	-	-	-	-	1.30	-	0.33	-	a & b
78	32.61	1798	(*Z*)-Hexadec-7-enal	-	-	-	1.82	-	-	-	-	-	-	a & b
79	33.05	1835	Hexadecanal	-	-	-	-	-	-	-	-	0.32	0.41	a & b
80	33.07	1863	Phthalic acid	-	2.39	-	-	1.06	0.82	-	-	-	-	a & b
81	33.98	1868	Bis(2-methylpropyl) benzene-1,2-dicarboxylate (Diisobutyl phthalate)	-	-	-	-	-	-	-	-	-	0.47	a & b
82	34.06	1877	4-Nonylphenol	-	-	-	-	-	-	-	-	0.38	-	a & b
83	34.2	1892	Nonadec-1-ene	-	-	-	-	-	-	-	-	0.25	-	a & b
84	34.46	1900	Nonadecane	-	-	2.16	-	-	-	0.29	-	-	-	a & b
85	38.99	1922	Dibutyl benzene-1,2-dicarboxylate	-	-	-	-	-	-	-	-	2.32	1.98	a & b
86	39.25	1926	Methyl hexadecanoate	**2.71**	**6.69**	0.73	1.30	1.87	1.04	-	-	-	0.34	a & b
87	40.00	1931	3-Methyl-2-(3,7,11-trimethyldodecyl)furan	-	-	-	-	-	-	-	-	0.31	0.41	a & b
88	40.09	1975	Heptadecan-1-ol	-	-	-	-	-	-	0.42	0.49	-	-	a & b
89	40.46	1995	(*Z*)-Octadec-9-enal (Olealdehyde)	-	-	-	-	-	-	-	-	-	1.04	a & b
90	41.38	2000	Icosane	-	-	-	-	-	-	**12.68**	0.21	-	-	a & b
91	42.46	2076	Methyl (9*Z*,12*Z*)-octadeca-9,12-dienoate	3.20	1.22	-	-	1.99	-	-	-	0.99	0.38	a & b
92	44.64	2081	Octadecan-1-ol	-	-	-	-	-	-	0.36	-	-	-	a & b
93	46.45	2086	(*Z*)-Octadec-9-enoic acid	-	-	-	-	-	3.15	-	-	-	-	a & b
94	46.63	2093	Methyl (7*E*,10*E*)-octadeca-7,10-dienoate	-	-	3.65	0.65	1.99	1.82	-	-	0.79	1	a & b
95	46.83	2100	Henicosane	-	-	-	-	-	-	**61.07**	**6.71**	-	-	a & b
96	47.16	2102	(*E*)-Octadec-2-enoic acid	1.29	-	-	-	-	-	-	-	-	-	a & b
97	47.38	2104	2-O-heptan-4-yl 1-O-(2-methylpropyl) benzene-1,2-dicarboxylate (Phthalic acid, hept-4-yl isobutyl ester)	-	-	-	-	-	-	-	-	1.56	1.19	a & b
98	47.52	2106	5-Dodecyloxolan-2-one	-	-	-	-	-	-	-	-	2	1.13	a & b
99	47.63	2108	Methyl (9*Z*,12*Z*,15*Z*)-octadeca-9,12,15-trienoate	-	-	-	-	6.04	-	-	-	-	-	a & b
100	47.73	2114	(*E*)-3,7,11,15-Tetramethylhexadec-2-en-1-ol	-	-	-	-	-	-	-	-	-	0.36	a & b
101	47.83	2116	(*Z*)-octadec-11-enoic acid (*cis*-Vaccenic acid)	-	-	-	-	-	-	1.80	-	-	-	a & b
102	47.92	2135	(8*Z*,11*Z*,14*Z*)-icosa-8,11,14-trienoic acid ((*Z*,*Z*,*Z*)-8,11,14-Eicosatrienoic Acid)	-	-	-	-	-	1.63	-	-	-	-	a & b
103	47.97	2159	Ethyl (9*Z*,12*Z*)-octadeca-9,12-dienoate (Ethyl linoleate)	-	-	-	-	-	0.91	-	-	-	-	a & b
104	48.6	2161	(*Z*)-octadec-9-enoic acid (Oleic acid)	-	-	-	-	-	-	0.79	-	-	-	a & b
105	48.68	2173	(9*Z*,12*Z*)-octadeca-9,12-dienoic acid (Linoleic acid)	-	-	-	-	-	-	0.73	-	-	-	a & b
106	49.69	2182	Hexadeceneamide	-	-	-	-	-	-	-	-	2.68	-	a & b
107	51.57	2200	Docosane	-	-	-	-	-	-	0.34	-	-	-	a & b
108	52.2	2231	Methyl (5*Z*,8*Z*,11*Z*,14*Z*)-icosa-5,8,11,14-tetraenoate (Methyl arachidonate)	-	-	-	-	-	-	-	-	**14.25**	**12.52**	a & b
109	52.45	2300	Tricosane	-	0.51	-	1.53	**10.51**	1.64	-	1.43	-	-	a & b
110	52.69	2319	3,7,11,15-Tetramethylhexadec-1-yn-3-ol	-	-	-	-	-	-	0.52	-	-	-	a & b
111	54.79	2321	Icosanoic acid (Arachidic acid)	1.22	-	**11.05**	**1.14**	2.90	2.30	-	0.84	**9.81**	-	a & b
112	54.94	2375	(*Z*)-octadec-9-enamide ((*Z*)-9-Octadecenamide)	-	-	-	-	-	-	-	-	**19.13**	**3.92**	a & b
113	55.2	2405	Methyl (9*E*,12*E*)-octadeca-9,12-dienoate	-	-	-	-	1.30	-	-	1.42	-	-	a & b
114	55.5	2500	Pentacosane	-	-	-	-	-	-	-	-	-	3.45	a & b
115	55.96	2663	2-Methylhexacosane	-	-	0.71	-	-	-	-	-	-	-	a & b
116	56.46	2700	Heptacosane	-	-	-	-	-	-	-	-	1.15	-	a & b
117	57.84	2889	2-Phenylethyl octadecanoate	-	-	-	1.29	**4.41**	**3.35**	-	**2.67**	**6.82**	**3.70**	a & b
118	58.77	3100	Hentriacontane	0.68	-	-	-	-	1.44	1.68	-	-	-	a & b
119	59.75	3200	Dotriacontane	-	-	-	-	-	-	-	-	-	0.53	a & b
120	60.14	3942	heptatriacontan-1-ol	-	-	-	-	-	-	-	-	0.94	0.38	a & b
**Monoterpenes**				-	**1.18**	-	**3.41**	-	-	-	**7.83**	**11.67**	**1.56**	
**Sesquiterpenes**				**72.68**	**76.06**	**62.82**	**65.23**	**54.24**	**74.07**	**13.56**	**65.14**	**18.21**	**39.3**	
**Diterpenes**				**0.79**	-	**9.52**	**17.64**	**5.79**	**5.68**	**1.40**	**6.59**	**0.52**	**17.63**	
**Carotenoid derived compounds**				**8.41**	**6.86**	-	**4.48**	-	-	-	**1.87**	**0.46**	-	
**Nonterpenoids**				**10.64**	**14.46**	**21.65**	**9.23**	**32.07**	**18.68**	**82.37**	**16.11**	**64.25**	**33.53**	
**Total identified**				**92.52**	**98.56**	**93.99**	**99.99**	**92.1**	**98.43**	**97.33**	**97.54**	**95.11**	**91.7**	

RT: retention time, KI: Kovat′s index determined experimentally relative to C8–C28 *n*-alkanes, HD: hydrodistillation, MAE: microwave-assisted extraction. a: The compounds were identified via AMDIS software (www.amdis.net (accessed on 29 January 2021)); b: The compounds were identified via Wiley spectral library collection and NIST library databases as well; c: The compounds were identified by a comparison with authentic standards.

**Table 2:** Minimal inhibitory concentration (MIC-µg/mL) for plant essential oils determined by microdilution assay.

Plant	Extraction Method	Bacteria		Fungi
		Gram-Positive	Gram-Negative	
		*S. aureus* ATCC29213	*E. coli* ATCC25922	*A. niger* NRC53
***C. scoparia***	MAE	1000.00	1000.00 NIE	NIE
	HD	125.00	1000.00	NIE
***C. calcitrapa***	MAE	31.25	500	NIE
	HD	1000.00	NIE	NIE
***C. glomerata***	MAE	31.25	500.00	NIE
	HD	31.25	1000.00	NIE
***C. lipii***	MAE	31.25	1000.00	1000.00
	HD	125.00	NIE	NIE
***C. alexandrina***	MAE	125.00	NIE	NIE
	HD	31.25	500.00	NIE
**Thiophenicol**		32	50	
**Treflucan**				64

NIE: noninhibitory effect.

## Data Availability

Not available.
